# Prevalence of Human Papillomavirus in 45 Greek Patients with Oral Cancer

**DOI:** 10.1155/2013/756510

**Published:** 2013-02-27

**Authors:** Maria Kouvousi, Dimitra Xesfyngi, Elpida Tsimplaki, Elena Argyri, Georgia Ioannidou, Maria Ploxorou, Andreas C. Lazaris, Efstratios Patsouris, Efstathia Panotopoulou

**Affiliations:** ^1^Department of Virology, “G. Papanikolaou” Research Center of Oncology and Experimental Surgery, Regional Anticancer Oncology Hospital of Athens “St. Savvas”, 171 Alexandras Avenue, 11522 Athens, Greece; ^2^Radiotherapy Department, Regional Anticancer Oncology Hospital of Athens “St. Savvas”, 171 Alexandras Avenue, 11522 Athens, Greece; ^3^1st Department of Pathology, Medical School, University of Athens, Goudi, 11527 Athens, Greece

## Abstract

The relation between HPV and head and neck cancer has recently and extensively been investigated. The purpose of this study was to indentify HPV genotypes, as well as E6/E7 mRNA expression of high-risk HPVs (16, 18, 31, 33 and 45) in oral squamous cell carcinomas (OSCCs) from 45 Greek patients. The overall prevalence of HPV DNA positive OSCCs was 11.1% (5/45), while high-risk HPV DNA was found in 6.7% (3/45) of OSCCs. E6/E7 mRNA expression was detected in 8.9% (4/45) of the oral cavity samples. Our data indicated that HPV 16 was the commonest genotype identified in HPV-positive OSCCs by both DNA and RNA tests. This study confirms the prevalence of HPV infections among patients with OSCCs. Future analysis and followup of more OSCCs will enable us to correlate HPV detection and clinical outcome.

## 1. Introduction

Cervical infection by one of approximately 15 high-risk HPV types is generally accepted as the necessary causative agent of cervical cancer [1, 2]. The upper aerodigestive tract, including the oral cavity, the pharynx, and the larynx, is also lined by a squamous mucous membrane and because of the morphological similarities and epitheliotropic nature of HPV, a link between head and neck squamous cell carcinoma (HNSCC) and HPV seems logical [3].

HNSCC is usually etiologically linked to tobacco and/or alcohol or other lifestyle habits, but a minority of patients develop HNSCC in the absence of exposure to these factors or any other obvious predisposing genetic defect. Several investigators have reported detection of HPV infection in healthy oral mucosa as well as in squamous cell carcinoma of the oral cavity (OSCC) and oropharynx (OPSCC) [4, 5], which are related to sexual behaviour and younger age [6]. HPV has shown to have a predilection for certain, especially nonkeratinized, anatomical sites of the oropharynx. High-risk HPV types, such as HPV 16, have been detected even in clinically normal oropharyngeal mucosa [4].

In the cervix, the persistence of HPV infection may increase the likelihood of viral integration and concomitant deregulation of viral protein expression, leading to overexpression of E6/E7 oncoproteins [7, 8]. In oral cancers the integration of HPV DNA into the host's cell genome is probably a less common event [9]. Various HPV types have been identified in patients with oral cancer [10], but clinical and case control studies have not proved a causal relationship between the virus and oral carcinomas beyond any reasonable doubt [8, 9, 11]. HPV DNA detection alone is regarded as insufficient evidence for a causal role in oral cells transformation and is more like a secondary invader [7, 10]. HPV integration into the host cell genome however, is a causal factor in head and neck carcinogenesis [11–13]. In many reports, in which the HPV-positivity varies from 40 to 57%, there is no reference to the viral integration of the HPV positive samples [5, 8, 10].

The purpose of our study is to define HPV detection in patients with OSCCs and to evaluate the detection of E6/E7 high-risk HPV mRNA as a possible biomarker for posttreatment surveillance.

## 2. Materials and Methods

Sixty-four (64) cytological samples were obtained from patients with HNSCC of the oral cavity, (35 oral tongue, 8 maxilla, 4 mandible, 8 floor of mouth, 6 retromolar trigone, and 3 buccal mucosa) who visited the outpatient clinic of the Radiotherapy Department of the Regional Anticancer Oncology Hospital “Saint Savvas”, Athens, Greece, between November 2008 and November 2011. Patients were eligible if they had not received any previous cancer treatment. Samples were obtained upon diagnosis. Ethical approval was granted by the ethics committee of Regional Anticancer Oncology Hospital of Athens “St. Savvas,” and all participants provided written, informed consent. The samples were collected using a brush and swabbing the mouth, maxilla and mandible, oral tongue, and buccal mucosa and were preserved in Thin Prep (PreservCyt Solution, Hologic UK).

### 2.1. Extraction of Nucleic Acids

Cytological samples were transferred in lysis buffer (NucliSENS lysis buffer, bioMérieux Hellas S.A, cat no. 200292) for 30 minutes, then total nucleic acid was extracted by the offboard protocol with the NucliSENS easyMAG platform (bioMérieux Hellas S.A), according to the manufacturer's instructions. The nucleic acids were eluted in 55 *μ*L of elution buffer. DNA quality test was carried out using Human Globin, Beta, Primer set kit (Maxim Biotech, Inc., South San Francisco, CA, USA) according to manufacturer's instructions. To assess RNA integrity, 5 *μ*g of RNA per sample was separated on 1% formaldehyde-agarose gel.

### 2.2. HPV Genotyping

Then, the PapilloCheck HPV-Screening (Greiner, Germany) was used for the type-specific identification of 24 types of HPV (15 high-risk types: 16, 18, 31, 33, 35, 39, 45, 51, 52, 56, 58, 59, 68, 73, and 82, probable high-risk types: 53, 66 and 7 low-risk types: 6, 11, 40, 42, 43, 44/55, and 70). This technology is based on a DNA chip for the type-specific identification of 24 types of HPV. E1-based PCR was performed according to the manufacturer's guidelines. For each sample, we mixed 19.8 *μ*L PapilloCheck MasterMix, 0.2 *μ*L HotStarTaq plus DNA polymerase (5U/*μ*L, Qiagen, cat no. 203605), and 5 *μ*L DNA from the tissue sample. Hybridization is followed by mixing 30 *μ*L of the PapilloCheck hybridization buffer in a new reaction tube with 5 *μ*L of the PCR product at room temperature and transferring 25 *μ*L of the hybridization mix into each compartment of the chip. We incubated the chip for 15 minutes at room temperature in a humid atmosphere. The chip was washed in 3 washing solutions, centrifuged for 3 minutes at 5000 rpm, and scanned on the CheckScannerTM.

### 2.3. HPV E6/E7 mRNA Expression

A commercial real-time NucliSENS EasyQ assay (NucliSENS EasyQ HPV 1.1, bioMérieux Hellas S.A, cat no. 290003) was performed for the qualitative detection of HPV E6/E7 mRNA of five high-risk HPV types (16, 18, 31, 33, and 45) according to the manufacturer's instructions. Firstly, three premixes were made by adding reagent sphere diluent (Tris-HCl, 45% DMSO) into reagent spheres (nucleotides, dithiotreitol, and MgCl_2_). In each premix, we added U1A/HPV 16, HPV 33/45, or HPV 18/31 primer and molecular beacon mixes, KCl stock solution, and NASBA water. Secondly, 10 *μ*L of this premix was distributed to each well in a reaction plate, and the addition of 5 *μ*L RNA followed. The plates were incubated for 4 minutes at 65°C to destabilize secondary structures of RNA, followed by cooling down to 41°C. The reaction was started by addition of enzymes (AMV-RT, RNase H, T7 RNA polymerase, and bovine serum albumin) and measured in real time using the Lambda FL 600 fluorescence reader (Bio-Tek, Winooski, VT) at 41°C for 2 hours and 30 minutes.

### 2.4. Treatment Procedures

Following diagnosis and molecular analysis, patients with positive histology of HNSCC of the oral cavity, with a Karnofsky Index (KI) ≥ 70%, and with normal renal, hepatic and bone marrow function were included. Baseline laboratories studies requirements included neutrophils greater than 1.5 × 10^9^/L and platelets >100 × 10^9^/L. Exclusion criteria were distant metastatic disease, a life expectancy of < 3 months, pregnancy, previous cancer disease within 5 years of study entry, existence of second primary tumor, and Karnofsky Index (KI) OF < 70%. Furthermore, patients with severe renal, liver, cardiovascular, or pulmonary diseases and patients with hematological malignancies were excluded.

All participants underwent an extensive presurgical evaluation that included clinical examination, panendoscopy, complete blood count and biochemical profile, clearance creatinine, cardiovascular examination, chest X-ray or CT, and CT and/or MRI of head and neck. 

Patients with early stage resectable disease (stage I-II) and selected patients of stage III (T_2_N_1_M_0_) were treated surgically. Patients with locally advanced disease (unresectable or marginally resectable), stage III-IVA, were treated either with induction chemotherapy or radical chemoradiation. Depending on response after induction chemotherapy, patients underwent either surgery followed by postoperative chemoradiation or radical combined radiation therapy with chemotherapy.

Before induction chemotherapy the palpable edges of the primary lesion (both the longest and the shortest axes) were marked by at least four points, which were 0.5 cm away. 

Tumor response was evaluated after the end of induction chemotherapy using a clinical examination and CT or MRI of the head and neck with tumor volumetry. Clinical tumor response to chemotherapy was defined by the RECIST criteria. Complete response (CR) was defined as a reduction of the tumor volume ≥ 70% up to the complete disappearance. Partial response (PR) was defined as at least a 30% decrease in the sum of diameters of tumor, taking as reference the baseline sum diameters. Progressive disease (PD) was defined as enlargement of tumor volume ≥ 20% or new tumor manifestations. Stable disease (SD) was defined as insufficient shrinkage to qualify for PR and insufficient increase to qualify for PD.

### 2.5. Outcome Measures

The median Follow-up interval was 18 months (range 4 to 36 months). Patients are monitored every two months in the first two years, every three to four months in the next 3 to 5 years, and once a year thereafter until death. Overall survival was calculated from the date of diagnosis to the date of death. Disease-free survival was estimated from the end of treatment to tumor recurrence, distant metastasis, or death from any cause.

### 2.6. Statistical Analysis

Data were analysed using SAS v9.0. Absolute and relative frequencies were used to present the HPV positivity. Chi-squared tests were performed to assess statistical significance of any differences in prevalence. 2 × 2 contingency tables Fisher's exact test was performed along with odds ratio and 95% confidence intervals calculation. *P* values of <0.05 were considered statistically significant.

## 3. Results

We collected 64 samples from the oral cavity, 19 of which were unsatisfactory for molecular analysis due to insufficient quantity of DNA, thus 45 samples were finally analysed (Table 1). The patients' ages ranged from 19 to 82 years (mean age: 51.9 years). 33 patients were male and 12 were female. Clinical, pathological, and treatment characteristics of the patients are described in Table 1. PapilloCheck assay detected high risk HPV DNA in 3/45 of the OSCCs (2 SCCs of the oral tongue and 1 of the floor of the mouth). The different HPV types revealed by the DNA and RNA test are shown in Table 2. In the 3 high-risk HPV DNA positive oral cavity cancers, HPV 16 was detected in 2 samples (1 case of SCC of the oral tongue and 1 of the floor of the mouth) and HPV 18 in 1 sample (1 case of oral tongue SCC). In addition, the low-risk HPV 6 was found in 2 oral tongue samples.

Further analysis using the NucliSENS EasyQ assay detected E6/E7 mRNA expression from the five high-risk HPV types (16, 18, 31, 33, and 45) in 4/45 of the total samples (8.9%) (3 cases of oral tongue SCC and 1 of the floor of the mouth). HPV 16 was the commonest type revealed by the RNA test as shown in Table 2. As far as the site-specific OSCC is concerned, HPV 16 was detected in 3 out of 5 HPV DNA positive oral cavity SCCs (2 cases of oral tongue SCC and 1 of floor of the mouth), while HPV 18 was found in 1 out of 5 HPV-infected oral cavity SCCs (1 case of oral tongue SCC), by the RNA detection method. 

## 4. Clinical Results

### 4.1. Definitive Surgery

8 of 45 patients (1 Stage I (T_1_N_0_M_0_), 5 Stage II (T_2_N_0_M_0_), 2 Stage III (T_2_N_1_M_0_)) underwent surgical excision (7 oral tongue, 1 maxilla). 4 of them had no adverse features, and they did not undergo any further treatment. The rest of the 4 patients with adverse features (>2 positive LN, resection margins <5 mm, perineural tumor invasion, and vascular space invasion) received external beam radiation therapy (EBRT) 54–60 Gy.

All patients who were treated surgically are so far disease-free (7 HPV DNA negative and 1 HPV DNA positive (defined as absence of recurrence at the primary site, assessed by physical examination at every clinic visit and imaging studies at specified intervals). However, one of them developed a second primary tumor of the lung and so far is disease-free from both primaries (Table 2).

### 4.2. Definitive Chemoradiation

From the 37 patients with locally advanced (unresectable or marginally resectable) disease (stage III-IVA), 28 patients underwent induction chemotherapy with 2 cycles of TPF (docetaxel 75 mg/m^2^, (d1) Cisplatin 75 mg/m^2^ (d1), and 5-FU 500 mg/m^2^ (d1-d5) (referred as treatment protocol A) and 9 patients underwent radical external beam radiation therapy (EBRT: 68–72 Gy) with concurrent chemotherapy (Cisplatin) (d1–d22) and weekly cetuximab due to medical contraindications for induction chemotherapy with TPF (referred as treatment protocol B). 

From 28 patients treated with protocol A, 18 had complete response. 10 of them underwent radical surgery and 8 of them underwent radical radiotherapy with concurrent chemotherapy. The inception of postoperative radiotherapy was recommended not later than 8 weeks after surgery. Depending on histological findings, patients underwent EBRT 54–60 Gy with or without concurrent chemotherapy (Cisplatin 75 mg/m^2^ d1, d22) and weekly cetuximab. For the patients with high-risk features, such as R1 resection, extra capsular nodal spread, and vascular space invasion, perineural invasion total dose of 66 Gy was granted.

From 18 patients with complete response, 14 patients are today free of disease (3 HPV DNA positive (HPV 18, 16, 6), 11 HPV DNA negative), 2 died from lung metastasis (1 HPV DNA positive (HPV 16), 1 HPV DNA negative), and 2 are still alive with local recurrence (2 HPV DNA negative) (Table 2).

The rest of the 10 patients who were treated with protocol A had partial response (Table 1). All of them underwent radical EBRT (68–72 Gy) with concurrent chemotherapy (Cisplatin) (d1–d22) and weekly cetuximab. All of them were HPV DNA negative. 1 of them is free of disease, 6 died from metastatic disease, 1 died from other causes, and 2 of them are still alive with local recurrence.

From 9 patients with stage III-IVA who were treated with protocol B, 5 of them are free of disease, 1 died from metastatic disease, and 3 are still alive with local recurrence. All of them were HPV DNA negative. 

## 5. Discussion

Of the approximately 120 HPV types so far known to infect the mucosal surfaces of the genital tract, 14 are considered to be “high risk” or “oncogenic” [1, 2]. Some of these high-risk types have been found in the oral cavity and oropharynx of both cancer-free adult individuals and in patients suffering from HNSCC [4, 7, 8, 12]. HPV 16, the HPV type most prevalent in cervical SCCs, is also the most common type present in HPV-positive HNSCCs [10, 13–15].

The great variation in HPV prevalence found in OSCCs in different studies may be due to differences among the analyzed population, but also due to differences in the samples tested (i.e., formalin-fixed or fresh biopsies, exfoliated fresh cells), the methods of DNA extraction, and, most importantly, the HPV detection methods used [8, 16]. Several studies using a variety of techniques, including immunohistochemistry, in situ hybridization, dot blot and Southern blot hybridization, and polymerase chain reaction (PCR), have been used to demonstrate the presence of HPV genome in HNSCC [4, 5, 7, 15].

Our study shows that the use of DNA and RNA detection methods confirms the prevalence of HPV infection among patients with OSCCs. HPV 16 infection has been found in 2 cases. In both cases infected by HPV 16, the E6/E7 transcripts have been detected by the RNA test. Coinfections were not completely absent. In one case with HPV 6 infection, what is interesting is that the RNA test revealed HPV 16 E6/E7 mRNA. This may be explained by the fact that total viral DNA of HPV 16 has been integrated into the host genome, and, therefore, it cannot be detected by the DNA test.

In 75% of the HPV-positive SCCs of the oral tongue, NucliSENS EasyQ assay detected E6/E7 mRNA expression of HPV 16 and HPV 18. HPV implication in oral tongue cancer has already been stated by many authors [5, 17]. 

On the other hand, clinical results indicated that the association between patient mortality and HPV detection was not statistically significant (*P* = 0.4215). The prevalence of HPV among currently disease-free patients was also not statically significant (*P* = 1.000).

As already stated, E6/E7 mRNA expression from high-risk HPV types is probably a less common event in HNSCC patients [13, 14]. Nevertheless different investigators' results suggest that HPV-positive carcinomas represent a different tumor entity [14, 18]. High-risk HPV types demonstrate an integration tendency, as can be assumed in our study by the mRNA oncoproteins' expression. We should also mention, as many investigators indicate, that low-risk HPV types (HPV 6 in our case) have been found in some head and neck carcinomas and might be implicated in the carcinogenesis process [14, 18, 19].

Although authors support better prognosis for HPV-positive oral cancer cases [3, 14, 16, 18], HPV-positive tumors are usually diagnosed in higher stage than HPV-negative tumors [20]. Therefore there is a small subgroup of HPV oral cancers that demonstrate a worse response to treatment and have a lower rate of survival [21].

Sexual transmission of oncogenic HPV genotypes is universally accepted for the anogenital region, whereas the route of infection for the oropharyngeal region is unclear [2, 18]. Although several studies have recently identified HPV, particularly type 16, in a subset of squamous cell carcinoma of the tonsils and base of the tongue [9, 14, 16, 21], the possibility of sexual or other transmission of HPV in oral cancer patients also needs to be explored [5, 6, 18]. The immune response might also affect the survival [3], but it seems to depend on whether HPV is present in the tumor cells in an episomal form or as an integrated virus [14, 18].

The tumor biology of HPV-positive OSCC is still under investigation [18]. HPV detection can be used as a prognostic factor or as a treatment strategy. Although our data showed a relatively low HPV and oral cancer association, people from different geographical regions often demonstrate higher or lower HPV prevalence in OSCC [22], which could also be the reason for our results. Despite the heterogeneity between different studies, HPV appears to play an important role in some types of oropharyngeal carcinomas and possibly a small subgroup of cancers in the oral cavity [23] and may represent an alternative pathway in carcinogenesis to the known and established factors of tobacco and alcohol [9, 18]

## 6. Conclusions

The ability to control head and neck cancer will therefore depend on three basic cornerstones: prevention, detection, and early diagnosis. The recent development of an HPV vaccine might offer hope for the prevention of cervical and anogenital carcinomas and possibly also represent an additional prevention option for a substantial number of patients with HNSCC.

This study described the detection rates and attribution of HPV genotypes as well as the E6/E7 mRNA expression of site-specific OSCCs in 45 Greek patients. HPV 16 accounted for most HPV-positive OSCCs. DNA and RNA assays detected the same genotypes in all high-risk HPV infected samples. This study confirms the prevalence of HPV infection among patients with OSCCs. Future analysis and followup of more OSCCs will enable us to correlate HPV detection and clinical outcome.

## Figures and Tables

**Table 1:** Patient, disease and medical treatment characteristics.

No. of patients	45
Age	
Median	51.9
Range	19–82
Gender	
Male	33
Female	12
Primary site	Oral cavity
Oral tongue	28
Floor of the mouth	6
Buccal mucosa	2
Maxilla	3
Mandible	1
Retromolar trigone	5
AJCC Stage	
I-II	7
III–IVA	38
DNA test	
Positive	5
Negative	40
Induction CMT^1^ (TPF)	
No	17
Yes	28
Response after CMT	
Complete	18
Partial	10

^1^CMT: Chemotherapy.

**Table 2:** Clinical outcome of site-specific OSCCs according to the HPV status.

Cancer origin	DNA test	RNA test	Clinical Results
Oral tongue SCC	HPV 16	HPV 16	Cancer free
Floor of the mouth SCC	HPV 16	HPV 16	Dead
Oral tongue SCC	HPV 18	HPV 18	Cancer free
Oral tongue SCC	HPV 6	HPV 16	Cancer free
Oral tongue SCC	HPV 6	negative	Cancer free
